# Compressed sensing real-time cine cardiovascular magnetic resonance: accurate assessment of left ventricular function in a single-breath-hold

**DOI:** 10.1186/s12968-016-0271-0

**Published:** 2016-08-24

**Authors:** Tomoyuki Kido, Teruhito Kido, Masashi Nakamura, Kouki Watanabe, Michaela Schmidt, Christoph Forman, Teruhito Mochizuki

**Affiliations:** 1 Department of Radiology, Ehime University Graduate School of Medicine, Shitsukawa, Toon, Ehime 791-0295 Japan; 2 Department of Radiology, Saiseikai Matsuyama Hospital, 880-2, Yamanishi, Matsuyama, Ehime 791-8026 Japan; 3 Department of Cardiology, Saiseikai Matsuyama Hospital, 880-2, Yamanishi, Matsuyama, Ehime 791-8026 Japan; 4 Siemens Healthcare GmbH, Allee am Roethelheimpark 2, 91052 Erlangen, Germany

**Keywords:** Cardiac function, Cardiovascular magnetic resonance, Compressed sensing, Left ventricular ejection fraction, Real-time imaging

## Abstract

**Background:**

Cardiovascular cine magnetic resonance (CMR) accelerated by compressed sensing (CS) is used to assess left ventricular (LV) function. However, it is difficult for prospective CS cine CMR to capture the complete end-diastolic phase, which can lead to underestimation of the end-diastolic volume (EDV), stroke volume (SV), and ejection fraction (EF), compared to retrospective standard cine CMR. This prospective study aimed to evaluate the diagnostic quality and accuracy of single-breath-hold full cardiac cycle CS cine CMR, acquired over two heart beats, to quantify LV volume in comparison to multi-breath-hold standard cine CMR.

**Methods:**

Eighty-one participants underwent standard segmented breath-hold cine and CS real-time cine CMR examinations to obtain a stack of eight contiguous short-axis images with same high spatial (1.7 × 1.7 mm^2^) and temporal resolution (41 ms). Two radiologists independently performed qualitative analysis of image quality (score, 1 [i.e., “nondiagnostic”] to 5 [i.e., “excellent”]) and quantitative analysis of the LV volume measurements.

**Results:**

The total examination time was 113 ± 7 s for standard cine CMR and 24 ± 4 s for CS cine CMR (*p* < 0.0001). The CS cine image quality was slightly lower than standard cine (4.8 ± 0.5 for standard vs. 4.4 ± 0.5 for CS; *p* < 0.0001). However, all image quality scores for CS cine were above 4 (i.e., good). No significant differences existed between standard and CS cine MR for all quantitative LV measurements. The mean differences with 95 % confidence interval (CI), based on Bland–Altman analysis, were 1.3 mL (95 % CI, −14.6 – 17.2) for LV end-diastolic volume, 0.2 mL (95 % CI, −9.8 to10.3) for LV end-systolic volume, 1.1 mL (95 % CI, −10.5 to 12.7) for LV stroke volume, 1.0 g (95 % CI, −11.2 to 13.3) for LV mass, and 0.4 % (95 % CI, −4.8 – 5.6) for LV ejection fraction. The interobserver and intraobserver variability for CS cine MR ranged from −4.8 – 1.6 % and from −7.3 – 9.3 %, respectively, with slopes of the regressions ranging 0.88–1.0 and 0.86–1.03, respectively.

**Conclusions:**

Single-breath-hold full cardiac cycle CS real-time cine CMR could evaluate LV volume with excellent accuracy. It may replace multi-breath-hold standard cine CMR.

## Background

Accurate and reproducible left ventricular (LV) volume assessment, in particular the ejection fraction (EF), is important in the management of various cardiac diseases because it is one of the strongest predictors of outcome [1–4]. Owing to high spatial and temporal resolution, a retrospective electrocardiogram (ECG)-gated breath-hold cine cardiovascular magnetic resonance (CMR) is generally the reference standard for assessing LV volume [5–8]. Standard cine CMR is well established; however, it requires multiple scans to cover the entire left ventricle for functional evaluation. Thus, this approach is prone to involve a prolonged CMR examination. In addition, it is difficult for critically ill patients to tolerate acquisitions involving multiple breath-holds and long examination times. To overcome this shortcoming of the cine CMR, various acceleration techniques have been developed [9–12]. The recent development of the compressed sensing (CS) technique with sparse sampling and iterative reconstruction promises to reduce drastically the acquisition time of CMR [13–16]. Accelerating cine CMR with the CS approach improves patient compliance, and enables a cine acquisition of the entire left ventricle in a single-breath-hold and eventually shorter examination time. Some studies have demonstrated the utility of CS cine CMR for evaluating LV function [17–20].

**Fig. 1:**
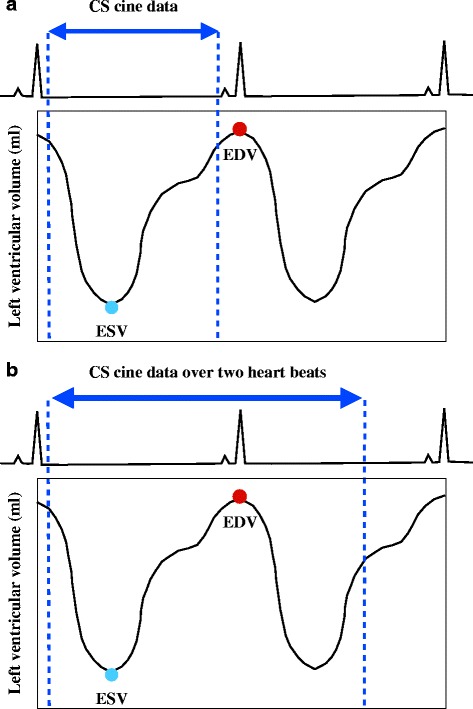
Data acquisition of compressed sensing cine CMR for LV volume measurements. *CS*, compressed sensing; *EDV*, end-diastolic volume; *ESV*, end-systolic volume

However, it is difficult for prospective ECG-triggered CS cine CMR to capture the complete end-diastolic phase in one heartbeat because it requires a finite time to detect the next ECG trigger [19, 20] as illustrated in Fig. 1a. When assessing LV function, this problem often leads to the underestimation of the end-diastolic volume (EDV), and accordingly the stroke volume (SV) and EF, compared to retrospective ECG-gated standard cine CMR [19]. To overcome this problem, we acquired the full cardiac cycle CS cine MR data over two heartbeats to capture the complete end-diastolic phase, which exists between the first and second heartbeat (Fig. 1b). The aim of this study was to evaluate the diagnostic quality and accuracy of single-breath-hold full cardiac cycle CS cine CMR for LV volume assessment in comparison to multi-breath-hold standard cine CMR.

## Methods

### Study population

This prospective study enrolled consecutive clinical patients with different cardiac conditions and healthy volunteers with no known cardiac disease. From August 2014 through May 2015, all participants underwent cine MR examinations, which included CS cine CMR and standard cine CMR sequences. All patients were clinically scheduled for CMR, and CS cine MR was part of the standard CMR protocol, as was standard cine CMR, which served as the standard of reference. The exclusion criteria for patient recruitment were arrhythmia and severely impaired breath-hold capacity.

### Cine magnetic resonance protocol

All CMR examinations were performed using a clinical 3 T MR scanner (MAGNETOM Skyra; Siemens Healthcare, Erlangen, Germany). Scout images were obtained to plan the cardiac axis views. The segmented balanced steady-state free-precession sequence was used for the retrospective ECG-gated standard cine CMR scans of all participants. The short-axis cine CMR images were obtained in a stack of eight contiguous slices spanning the entire left ventricle from the base to the apex. The prospective ECG-triggered CS real-time cine CMR scans using a prototype sequence were performed immediately after the standard cine CMR scans. All scans were performed at end-inspiratory. The temporal resolution, spatial resolution, and slice orientations were identical in the two cine protocols. Detailed imaging parameters are listed in Table 1. The scan time for full cardiac cycle CS cine CMR was three heartbeats per slice. While one heartbeat was used to obtain the magnetization steady state, the remaining two heartbeats were utilized for data acquisition covering the full cardiac cycle CS cine data.

**Table 1:** Imaging parameters

	Standard cine	CS cine
ECG gating	Retrospective	Prospective
TE/TR (ms)	1.4/3.2	1.4/3.2
FOV (mm)	350 × 350	350 × 350
Image matrix	208 × 166	208 × 166
Spatial resolution (mm)	1.7 × 1.7	1.7 × 1.7
Temporal resolution (ms)	41	41
Slice thickness (mm)	6	6
Flip angle	50	50
Bandwidth (Hz/pixel)	1145	960
Cardiac phases	25	19–31
Breath-holds (*n*)	4	1
Acceleration factor	3	12.8
Iterative reconstruction	—	80

*CS* compressed sensing, *ECG* electrocardiogram, *FOV* field of view, *TE* echo time, *TR* repetition time

### Data acquisition and image reconstruction of the CS real-time cine

**Fig. 2:**
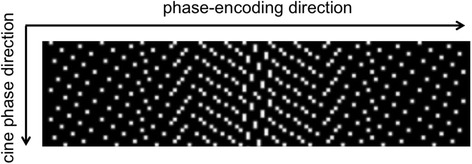
Sampling pattern of compressed sensing cine CMR

Data acquisition is performed using sparse, incoherent sampling of k-space. This is realized with a random distribution of the readouts on the Cartesian grid in k-space as illustrated in Fig. 2. Two parameters are used to adapt the sampling pattern, which are set to 7 and 13. While the first parameter defines the sub-sampling rate at k-space center, it increases towards the high frequencies to a sub-sampling rate defined in the second parameter. From frame-to-frame a random offset is applied which results in an incoherent temporal jitter.

Image reconstruction was performed with a non-linear, iterative SENSE-type approach implementing spatio-temporal regularization using redundant Haar wavelets as described in [21]. The corresponding cost function was solved with a Fast Iterative Shrinkage-Threshold Algorithm (FISTA) type optimization consisting of a gradient descend step for the quadratic terms and the evaluation of the proximal operator. The proximal operator is weighted with the regularization parameter, which was set to 0.001 and 0.005 for spatial and temporal regularization, respectively. The optimization was terminated after 40 iterations.

### Qualitative image quality analysis

Two radiologists with 7 years and 5 years of experience in cardiac imaging assessed all short-axis cine CMR images independently with focus on the clearness of myocardial border and artifact. The image quality of each cine CMR image was evaluated visually and scored on a five-point scale: 1 = nondiagnostic quality, extensive artifact affecting volumetric analysis, 2 = poor quality, moderate artifact affecting volumetric analysis, 3 = adequate quality, mild artifact affecting volumetric analysis, 4 = good quality, minimal not artifact affecting volumetric analysis, 5 = excellent quality, no artifact.

### Quantitative LV volume analysis

For quantitative measurements, the stack of eight contiguous short-axis slices of both cine CMR images was assessed independently by the two radiologists using the dedicated software package SYNAPSE VINCENT (Fujifilm Corp., Ltd, Tokyo, Japan). The epicardial and endocardial contours were automatically traced on the short-axis images. Contours rendered by automated analysis were reviewed and manually corrected, as necessary. The endocardial trabeculations and papillary muscles of the left ventricle were included in the LV cavity volumes [22–24]. The most basal slice with at least a semicircular muscular ring at the end-systolic phase was regarded as the base, and the most apical slice with a visible cavity at end-diastolic phase was regarded as the apex [25]. The LV volume and LV mass were calculated using the Simpson method. The end-systolic and end-diastolic phases were detected automatically by software, based on the smallest and largest LV volumes over the entire cardiac cycle.

### Statistical analysis

The continuous data are expressed as the mean ± the standard deviation (SD) or as the median (first quartile, third quartile), as appropriate, based on distribution. The Wilcoxon matched-pairs signed-rank test was used to compare image quality between standard cine CMR and CS cine CMR. The interobserver agreement on image quality was determined using the *kappa* test. The paired *t* test was used to compare the scan time. The results of EDV, end-systolic volume (ESV), SV, LV mass, and EF on standard cine CMR and CS cine CMR were compared with the Wilcoxon matched-pairs signed-rank test. Linear regression and Bland–Altman analysis were used to evaluate the correlation and agreement between these LV measurements. In addition, interobserver and intraobserver variabilities in CS cine CMR were also determined by the same analysis. A *p* value of less than 0.05 was considered statistically significant. All statistical analyses were performed by commercially available software (JMP version 11; SAS Institute, Cary, NC, USA). Sample size calculation was based on the primary outcome of the difference between the EF measures obtained from the two cine methods. Seventy-four participants were calculated to provide 80 % power to detect more than 5 % absolute difference in EF measures with a two-sided significance level of 0.05, assuming a common SD for the mean EF measurement of 15 %. The EF margin was considered the clinically acceptable range, based on previous research [26–30]. We ultimately enrolled 90 participants with the expectation of 18 % attrition.

## Results

Among the 90 enrolled participants, six patients with arrhythmia and three patients with severely impaired breath-hold capacity were excluded from the study. Eighty-one participants (65 patients and 16 volunteers) were ultimately used for qualitative analysis of image quality and for quantitative analysis of LV volume measurements. All 81 participants had a regular sinus rhythm with the mean heart rate of 62 ± 10 bpm (range, 42–88 bpm) during both cine CMR scans. The total examination time was 113 ± 7 s (range, 100–130 s) for standard cine MR and 24 ± 4 s (range, 16–34 s) for CS cine MR (*p* < 0.0001).

### Image quality

**Fig. 3:**
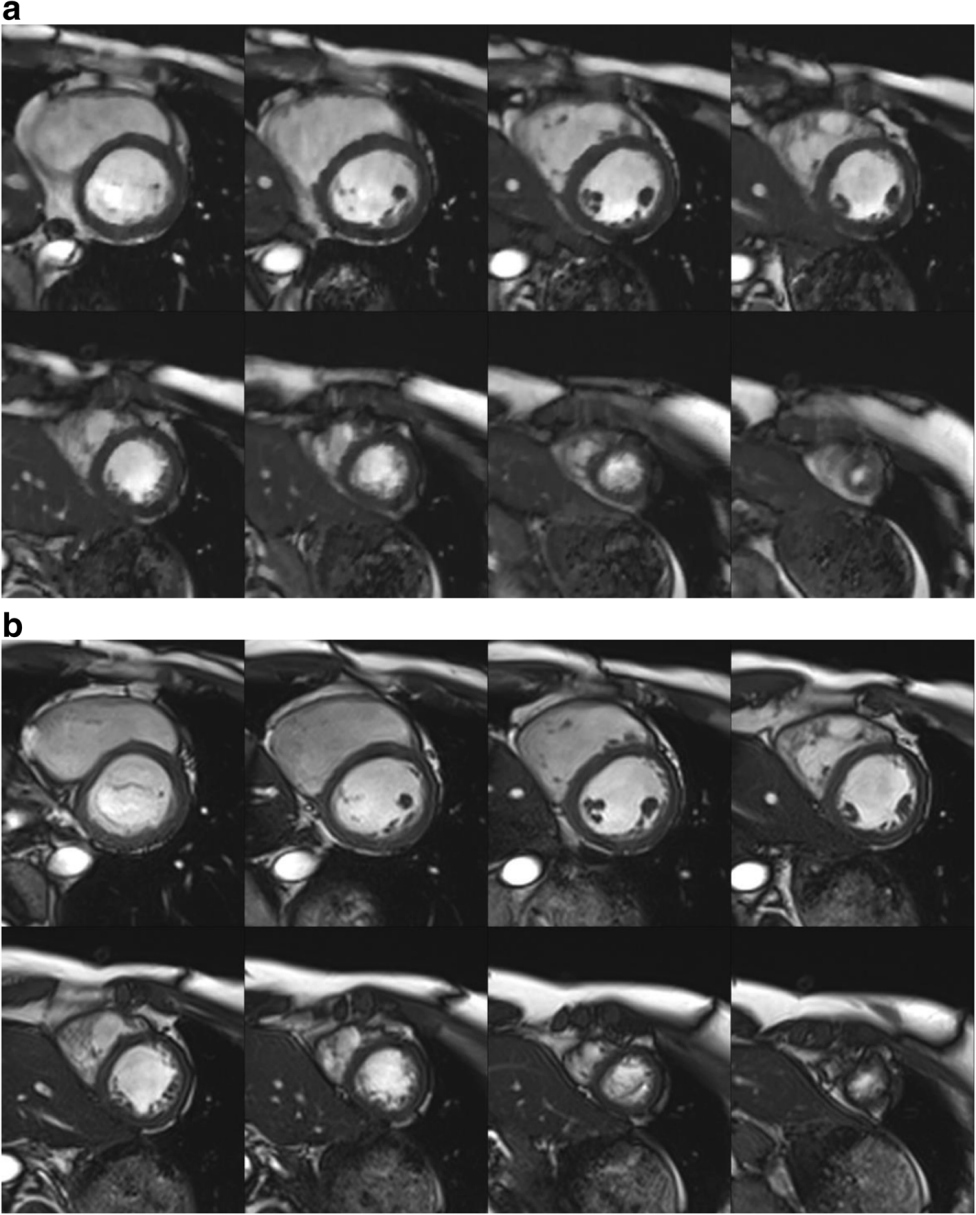
Images acquired using compressed sensing cine and standard cine CMR. End-diastolic short-axis views of the left ventricle **a** by compressed sensing cine CMR and **b** by standard cine CMR. Both image sets were acquired from a 29-year-old healthy male volunteer. Both observers rated the image quality as excellent (i.e., score 5) for both images

Figure 3 shows representative sets of standard cine CMR and CS cine CMR images in eight short-axis slices from one healthy volunteer. Both cine CMR images showed excellent diagnostic image quality. The CS real-time cine CMR yielded slightly worse image quality scores than standard cine CMR (4.8 ± 0.5 for standard vs. 4.4 ± 0.5 for CS; *p* < 0.0001). There was good interobserver agreement of image quality for standard cine CMR (*kappa* score = 0.82) and for CS cine CMR (*kappa* score = 0.80).

### Left ventricular function

**Fig. 4:**
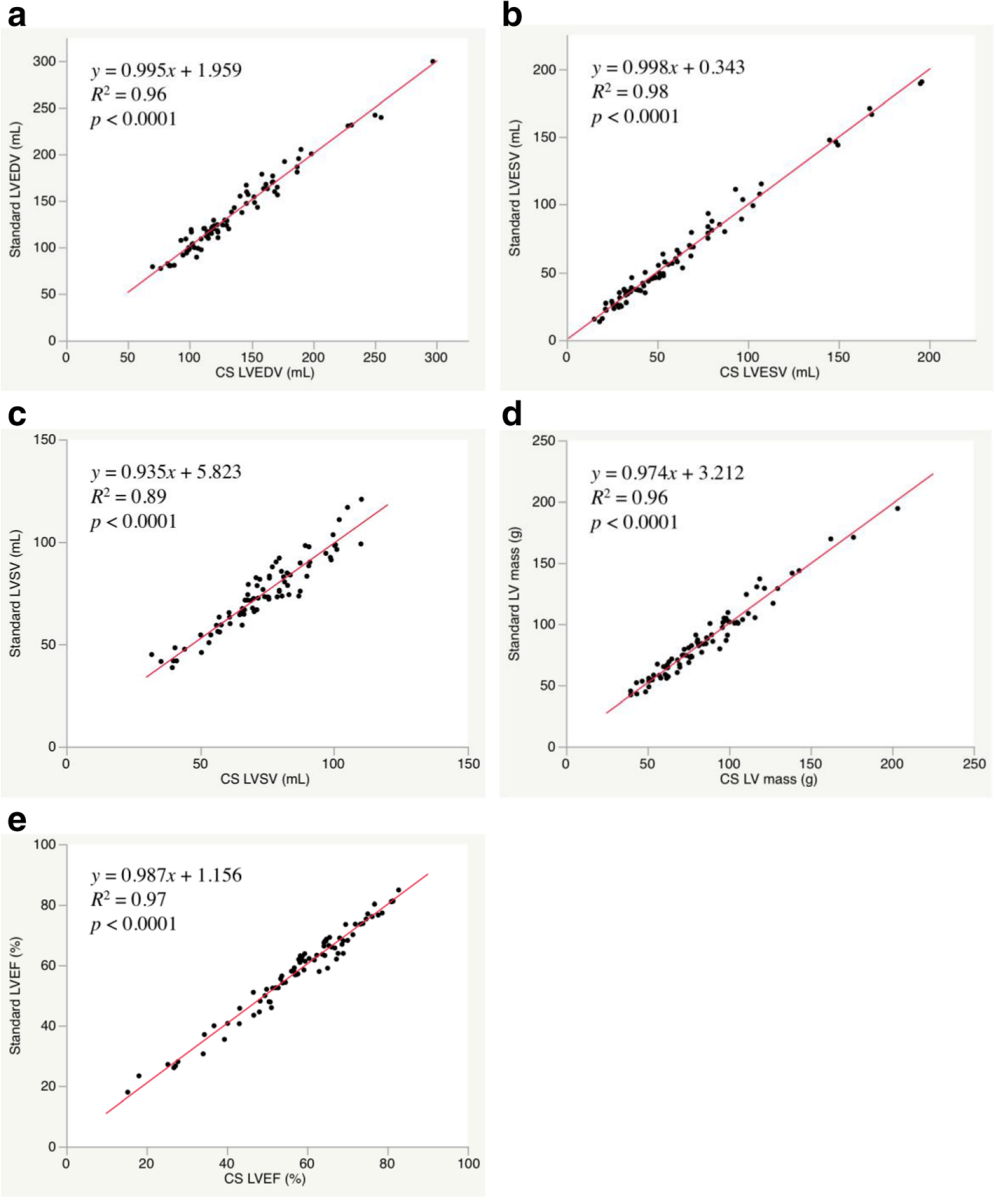
Scatter plots for LV volume measurements by standard cine and CS cine. *CS*, compressed sensing; *EF*, ejection fraction; *LV*, left ventricular; *LVEDV*, left-ventricular end-diastolic volume; LVEF, left ventricular ejection fraction; *LVESV*, left ventricular end-systolic volume; *LVSV*, left-ventricular stroke volume; *SD*, standard deviation

**Fig. 5:**
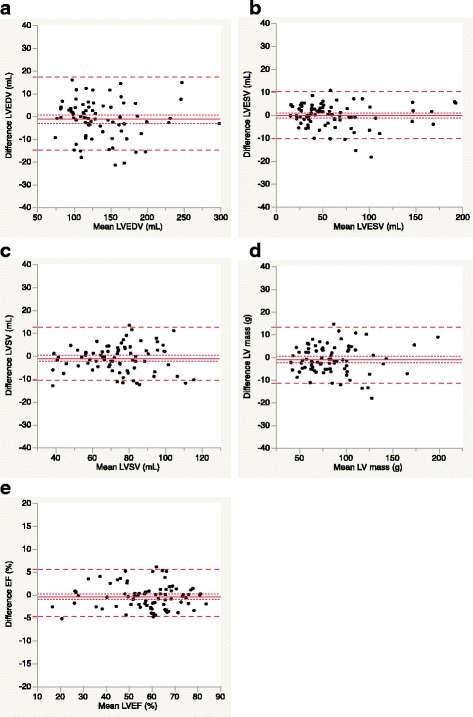
Bland–Altman plots for LV volume measurements by standard cine and CS cine. The solid line indicates the difference between two sequences; the long dashed lines indicate the 95 % limits of agreement interval (i.e., the mean ± 1.96 SD); and the short dashed lines indicate the 95 % confidence interval of the mean difference *CS*, compressed sensing; *EF*, ejection fraction; *LV*, left ventricular; *LVEDV*, left-ventricular end-diastolic volume; LVEF, left-ventricular ejection fraction; *LVESV*, left-ventricular end-systolic volume; *LVSV*, left-ventricular stroke volume; *SD*, standard deviation

All 81 standard and CS cine CMR images showed good to excellent image quality and they were sufficient to undergo quantitative analysis of the LV volume (Table 2). Multi-breath-hold standard cine CMR images were used as the standard reference for LV volume measurements (i.e., LVEF, LVEDV, LVESV, LVSV, and LV mass). Table 3 shows the median (first quartile, third quartile) values for the volumetric analysis of LV volume measurements and the analysis of the respective differences between standard and CS cine MR. There were no significant differences between standard and CS cine CMR for all LV volume measurements. The linear regression yielded good agreement between standard and CS cine CMR for all measurements (Fig. 4). Bland–Altman analysis revealed that the mean difference with 95 % confidence interval (CI) between the standard cine CMR and the CS cine CMR were 1.3 mL (95 % CI, −14.6 mL to 17.2 mL) for LVEDV, 0.2 mL (95 % CI, −9.8 mL to10.3 mL) for LVESV, 1.1 mL (95 % CI, −10.5 mL to 12.7 mL) for LVSV, 1.0 g (95 % CI, −11.2 g to 13.3 g) for LV mass, and 0.4 % (95 % CI, −4.8 – 5.6 %) for LVEF (Fig. 5). The interobserver and intraobserver variability for CS cine CMR ranged from −4.8 – 1.6 % and from −7.3 – 9.3 %, respectively, with the slopes of regression ranging 0.88 –1.0 and 0.86–1.03, respectively (Table 4).

**Table 2:** Characteristics of the study population

	Patients	Volunteers
Number	65	16
Age (y)	70.9 ± 9.2	28.1 ± 4.4
Sex (female/male)	18/47	4/12
Height (m)	160.8 ± 9.5	166.9 ± 8.7
Weight (kg)	62.1 ± 13.2	60.6 ± 10.7
BMI (kg/m^2^)	23.8 ± 3.4	21.6 ± 2.2
HR (beats/min)	62.2 ± 10.3	62.1 ± 8.6
Cardiovascular risk factor		
Hypertension	36 (55 %)	—
Dyslipidemia	34 (52 %)	—
Diabetes mellitus	26 (40 %)	—
Smoking	16 (25 %)	—
Family history of CAD	9 (14 %)	—
Cardiovascular disease		
CAD	44 (68 %)	—
Cardiomyopathy	14 (22 %)	—
Valve disease	3 (5 %)	—
Other	4 (6 %)	—
LVEF <50 %	20 (31 %)	—

The data are presented as the mean ± standard deviation or as the median (first quartile, third quartile) or as the number (%) of subjects

*BMI* body mass index, *CAD* coronary artery disease, *HR* heart rate, *LV* left ventricular, *LVEDV* left ventricular end diastolic volume, *LVEF* left ventricular ejection fraction, *LVESV* left ventricular end systolic volume, *LVSV* left ventricular stroke volume

**Table 3:** The LV volume measurements between standard cine and CS cine

	Standard cine (*n* = 81)	CS cine (*n* = 81)	*p*
LVEDV (mL)	121.0 (105.8, 161.7)	122.2 (103, 159)	0.28
LVESV (mL)	48.3 (34.8, 79.1)	50.9 (32.9, 77.8)	0.77
LVSV (mL)	73.5 (63.8, 85.2)	73.4 (63, 83.5)	0.15
LV mass (g)	82.3 (64.7, 101.3)	80.4 (62.1, 99.0)	0.15
LVEF (%)	61.3 (50.5, 68.0)	58.8 (50.7, 67.5)	0.10

The data are presented as the median (first quartile, third quartile)

*CS*, compressed sensing; *LV*, left ventricular; *LVEDV*, left ventricular end diastolic volume; *LVEF*, left ventricular ejection fraction; *LVESV*, left ventricular end systolic volume; *LVSV*, left ventricular stroke volume

**Table 4:** Intraobserver and interobserver variability of the CS cine CMR

	Difference (mean ± SD)	Variability [%] (mean ± SD)	*R* ^2^	Slope	*p*
Intraobserver					
LVEDV (mL)	0.3 ± 11.6	1.0 ± 4.7	0.98	1.0	<0.0001
LVESV (mL)	0.4 ± 3.8	1.1 ± 7.0	0.99	0.99	<0.0001
LVSV (mL)	0.2 ± 11.1	1.6 ± 7.7	0.91	0.92	<0.0001
LV mass (g)	−5.4 ± 9.4	−4.8 ± 8.8	0.95	0.88	<0.0001
LVEF (%)	0.1 ± 2.9	0.7 ± 4.9	0.97	0.96	<0.0001
Interobserver					
LVEDV (mL)	8.0 ± 5.8	6.4 ± 5.1	0.98	1.03	<0.0001
LVESV (mL)	1.7 ± 4.8	2.6 ± 9.8	0.99	1.03	<0.0001
LVSV (mL)	6.3 ± 6.0	9.3 ± 8.9	0.89	0.98	<0.0001
LV mass (g)	−6.3 ± 11.5	−7.3 ± 13.2	0.88	0.86	<0.0001
LVEF (%)	1.5 ± 3.4	2.9 ± 6.3	0.95	0.97	<0.0001

Bland–Altman plots highlight the mean difference and the standard deviation of the difference between the two measurements. Variability [%] is the absolute value of the difference between the two measurements divided by the mean of the two measurements

*CS* compressed sensing, *LV* left ventricular, *LVEDV* left ventricular end diastolic volume, *LVEF* left ventricular ejection fraction, *LVESV* left ventricular end systolic volume, *LVSV* left ventricular stroke volume, *MR* magnetic resonance, *SD* standard deviation

## Discussion

In this prospective study, single-breath-hold full cardiac cycle CS cine CMR showed high agreement for the volumetric analysis of the left ventricle, compared to the current reference standard multi-breath-hold cine CMR. Some previous studies have also shown that CS cine CMR is similar to standard cine CMR in image quality, and that the LV volume measurements were in good agreement. However, other investigators also report that CS cine CMR with prospective ECG-triggering cannot detect the very first and last phases of the cardiac cycle [19, 20]. This limitation often leads to the underestimation of EDV, SV, and EF. Our results suggested that single-breath-hold full cardiac cycle CS cine CMR could overcome this limitation by acquiring data over two heartbeats. In addition, an accurate assessment of LV function depends on spatial and temporal resolution [31]. Compromised spatial and temporal resolution often causes a substantial problem in image quality and in the accuracy of highly accelerated cine CMR in comparison to the standard cine CMR [32]. In our study, the high spatial (1.7 mm × 1.7 mm) and temporal (41 ms) resolutions of CS cine CMR were identical to those of standard cine CMR and translated into good image quality and high agreement for all LV measurements. Furthermore, we found that the variability of the LVEF (95 % CI, −4.8 – 5.6 %) between standard cine CMR and CS cine CMR measured in our study was comparable to the interstudy variability of LVEF measurements of standard cine CMR (95 % CI, −4.1 – 4.3 %) reported in previous research [8, 33]. These results indicate that the current single-breath-hold CS cine CMR was accurate and sufficiently reproducible to replace multi-breath-hold standard cine CMR. In addition, a rapid CS cine CMR examination is more cost-effective than multi-breath-hold standard cine CMR and particularly beneficial for ill patients who cannot tolerate prolonged examination times.

The CS cine CMR yielded a slightly worse image quality score, compared to standard cine CMR. Some CS cine CMR images had worse image quality because fold-over artifacts and flow-related artifacts occurred in the phase-encoding direction during the systolic phase. However, image quality scores were above 4 (i.e., good) for all CS cine CMR. This result suggested that acceptable diagnostic image quality could be achieved by CS cine CMR. Moreover, all patients with arrhythmia or impaired breath-hold capacity were excluded in this study because we were using the standard cine CMR images as the standard reference for LV measurements. In this study, this exclusion may have been advantageous in terms of image quality for standard cine CMR. These excluded patients often have image deterioration on the retrospective standard cine CMR image and impaired accuracy in the volumetric analysis in real clinical settings [34, 35]. On the other hand, the prospective CS real-time cine CMR has the advantage of being inherently insensitive to arrhythmia or respiratory motion because of the single-shot acquisition [36, 37]. In this study, the maximal number of cine CMR slices that a single-breath-hold can support will be limited by the ability of breath-hold. A variety of acceleration techniques could develop free-breathing cine CMR to overcome this limitation [38, 39]. The CS real-time cine CMR is also suited to be scanned in free-breathing as shown in another study [40]. In the future, further evaluation is needed to assess the clinical utility of the current CS cine CMR in free-breathing or in patients with arrhythmia.

### Limitations

**Fig. 6:**
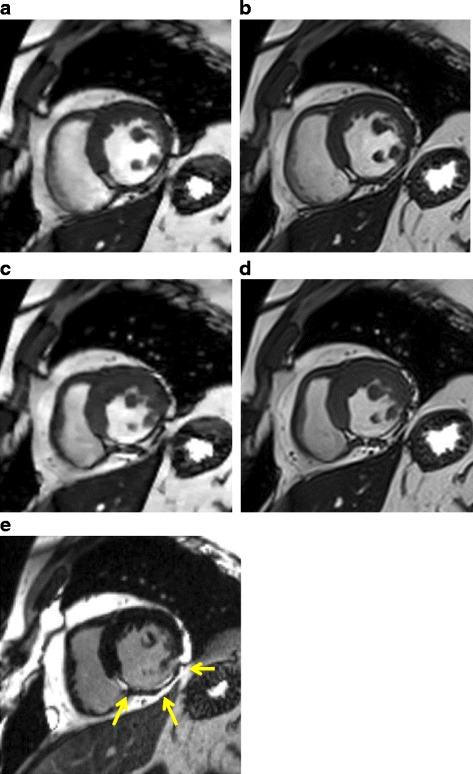
A 69-year-old patient with an inferior wall infarction. **a**, **c** Compressed sensing cine CMR images. **b**, **d** Standard cine MR images. **e** Late Gadolinium Enhancement (LGE) CMR images. The images depict the mid short-axis view **a** and **b** in the end-diastolic phase and **c** and **d** in the end-systolic phase. Both cine images show thinning and akinesia of the inferior myocardium. The LGE CMR shows a transmural infarction in the inferior myocardium (arrows)

The CS cine CMR is adequate for LV volume measurements; however, there are some limitations to this study. First, we did not evaluate regional myocardial wall motion abnormalities. This assessment is also important and a desired application of cardiac cine CMR in clinical practice. However, we could visually detect a regional myocardial wall motion abnormality of patients with myocardial infarction using the CS cine image and the standard cine image (Fig. 6). We expect that further examination will reveal the accuracy of CS cine CMR for assessing regional wall motion in patients with myocardial infarction. Second, the EF in this study group covered almost the entire range of clinical relevance (i.e., 18–85 %), but only 25 % of patients showed an EF less than 50 %. Third, we didn’t make an experimental study on effects of temporal and spatial resolution for CS reconstruction. We’ll need further consideration about the effect of higher temporal resolution than 41 ms for CS reconstruction.

Finally, in this study, the CS reconstruction was performed inline at the scanner at the end of the acquisition using CPU on the MR reconstruction system. The reconstruction time needed for the CS cine stack was approximately 3 min. The reconstruction time will increase proportionally with acquired slices and cine phases. Real-time visualization of cine CMR images is desirable for clinical utility. More computationally efficient CS reconstruction techniques, such as using the graphics processing unit [41], will reduce the reconstruction times and overcome this limitation in the future.

## Conclusions

The single-breath-hold full cardiac cycle CS real-time cine CMR can potentially replace the multi-breath-hold standard cine CMR. This technique will be beneficial for ill patients who cannot tolerate prolonged examination times or who have severely impaired breath-hold capacity.

## Abbreviations

CI, confidence interval; CS, compressed sensing; EDV, end-diastolic volume; EF, ejection fraction; ESV, end-systolic volume; LV, left ventricular; CMR, cardiovascular magnetic resonance; SD, standard deviation; SV, stroke volume.
